# Salicylic Acid and Melatonin Alleviate the Effects of Heat Stress on Essential Oil Composition and Antioxidant Enzyme Activity in *Mentha × piperita* and *Mentha arvensis* L.

**DOI:** 10.3390/antiox8110547

**Published:** 2019-11-13

**Authors:** Milad Haydari, Viviana Maresca, Daniela Rigano, Alireza Taleei, Ali Akbar Shahnejat-Bushehri, Javad Hadian, Sergio Sorbo, Marco Guida, Caterina Manna, Marina Piscopo, Rosaria Notariale, Francesca De Ruberto, Lina Fusaro, Adriana Basile

**Affiliations:** 1 Department of Agronomy and Plant Breeding, Collage of Agriculture and Natural Resources, University of Tehran, P.O. Box 31787-316, Karaj 77871-31587, Iran; milad.heydari@ut.ac.ir (M.H.); ataleei@ut.ac.ir (A.T.); ashah@ut.ac.ir (A.A.S.-B.); 2 Department of Biology—University of Naples “Federico II”, 80126 Naples, Italy; viviana.maresca@unina.it (V.M.); marco.guida@unina.it (M.G.); marina.piscopo@unina.it (M.P.); francesca.deruberto@gmail.com (F.D.R.); 3 Department of Pharmacy, School of Medicine and Surgery, University of Naples Federico II, 80126 Naples, Italy; drigano@unina.it; 4 Medicinal Plants and Drug Research Institute, ShahidBeheshti University, G.C. Tehran 11369, Iran; j_hadian@sbu.ac.ir; 5 C.e.S.M.A. University of Naples “Federico II”, 80126 Naples, Italy; sersorbo@unina.it; 6 Department of Precision Medicine, School of Medicine, University of Campania “Luigi Vanvitelli”, via Luigi de Crecchio, 80138 Naples, Italy; caterina.manna@unicampania.it (C.M.); notarialer@gmail.com (R.N.); 7 Department of Environmental Biology, Sapienza University of Rome, P.le Aldo Moro 5, 00185 Rome, Italy; lina.fusaro@uniroma1.it

**Keywords:** mentha, heat stress, antioxidant enzyme activity, salicylic acid, melatonin, essential oil

## Abstract

The aim of this study was to evaluate changes in the chemical profile of essential oils and antioxidant enzymes activity (catalase CAT, superoxide dismutase SOD, Glutathione *S*-transferases GST, and Peroxidase POX) in *Mentha × piperita* L. (Mitcham variety) and *Mentha arvensis* L. (var. *piperascens*), in response to heat stress. In addition, we used salicylic acid (SA) and melatonin (M), two brassinosteroids that play an important role in regulating physiological processes, to assess their potential to mitigate heat stress. In both species, the heat stress caused a variation in the composition of the essential oils and in the antioxidant enzymatic activity. Furthermore both Salicylic acid (SA) and melatonin (M) alleviated the effect of heat stress.

## 1. Introduction

The Lamiaceae family encompasses various genera, including aromatic herbs such as mint. Embracing half a dozen cultivated species, mint genus includes more than 30 species that are scattered worldwide, chiefly in temperate and tropical/subtropical regions. One of the distinctive features is that mint species possess essential oils [1]. 

Japanese mint or Cornmint (*Mentha arvensis* L. var. *piperascens* (Malinv. ex Holmes) Malinv. ex L.H.Bailey) is a fundamental natural source of monoterpenes, particularly L-menthol (up to 80% menthol), and it was already cultivated in ancient Japan as well as in China, India, and Brazil. 

*Mentha × piperita* is an abortive hybrid of the species *M. aquatica* L. and *M. spicata.* Ecumenically, peppermint is one of the most commercial odorous scented herbs. The peppermint leaves have not only a peculiar, sweet, and strong odor, but also a redolent, warm, and spicy taste, with a cooling aftertaste. The supremacy of the essential oils of *Mentha × piperita* is due to the presence of menthone, isomenthone, and different isomers of menthol. Nowadays, extensive usage of peppermint oil in flavoring chewing gums, sugar confectioneries, ice creams, desserts, baked goods, tobacco, and alcoholic beverages is just one of the most prevalent applications of such oils. Furthermore, it is also commonly employed in the flavoring of pharmaceutical and oral preparations [2].

Menthol shows various biological activities, such as sedative, anesthetic, antiseptic, gastric, and antipruritic. It is also one of the few natural monocyclic monoterpene alcohols that have characteristics conducive to fragrances. As such, it has been used to flavor various goods such as candies, chewing gums, and toothpaste [3,4].

Heat stress has effects on metabolite synthesis in aromatic plants, changing phenolic and antioxidants concentrations [5]. Heat stress induces the generation of reactive oxygen species (ROS), such as superoxide radicals (•O_2_^−^), hydrogen peroxide (H_2_O_2_), and hydroxyl radicals (•OH), in plants, thereby creating a state of oxidative stress in them. This increased ROS level in plants causes oxidative damage to biomolecules such as lipids, proteins, and nucleic acids, thus altering the redox homeostasis [6,7]. To avoid potential damage by ROS, a balance between production and elimination of ROS at the intracellular level must be regulated. This equilibrium between production and detoxification of ROS is sustained by enzymatic and nonenzymatic antioxidants [8,9]. The enzymatic components comprise several antioxidant enzymes, such as superoxide dismutase (SOD), catalase (CAT), glutathione peroxidase (GPX), guaiacol peroxidase (POX), and peroxiredoxins.

Salicylic acid (SA) and melatonin (M) are two brassinosteroids playing an important role in regulating physiological processes. SA is a phenolic compound with antioxidant properties, involved in the regulation of physiological processes in plants [10]. SA can modulate plant responses to a wide range of oxidative stresses [11]. When applied exogenously at suitable concentrations, SA was found to enhance the efficiency of antioxidant system in plants [12]. M (N-acetyl-5-methoxytryptamine) is an indole hormone involved in multiple biological processes [13]. According to a lot of findings, M plays an important role in the regulation of plant growth and development [14] and provides a defense against abiotic stresses such as extreme temperature, excess copper, salinity, and drought [15,16,17]. A lot of studies have proven that M may act as a plant growth regulator in rooting, seed germination, and delay in leaf senescence and other morphogenetic features [18,19,20]. M has been observed to improve tolerance for multiple stresses including heat stress, and in particular, exogenous M treatments protect plants from temperature extremes [21].

The aim of this study was to evaluate the response of *M. arvensis* L. var*. piperascens* and *M. × piperita* to heat stress in relation to the production of essential oils in general and in particular of menthol, menthone, and isomenthone, which have considerable economic importance and play an important role in the industrial field. In particular, our goal was to investigate the potential of SA and M to mitigate the heat stress effects on the two plants, focusing on the variation of essential oils composition and antioxidant enzymes activity.

## 2. Materials and Methods

### 2.1. Plant Material, Culture, and Treatment

*M.* × *piperita* L. var. Mitcham and *M. arvensis* var. *piperascens* Malinv. ex L.H. Bailey were obtained from the “Safiabad agricultural and natural resources research and education center”. Planting and cultivation conditions were carried out in the growth chambers. The method is described in detail in Heydari et al. [22].

After 40 culturing days, plants were sprayed with SA (2, 3, and 4 mM, reported as SA2, SA3, and SA4, respectively) and M (10 and 30 M, reported as M1 and M3), together with M and SA at the highest concentrations (M3SA4 ). Tap water was used for controls.

For each treatment, we selected 50 sample plants for subsequent experiments.

The abbreviation used to antioxidant enzyme activity and GC and GC-MS (Gas chromatography - Mass spectrometry) analysis are:

MpH1C = *M.* x *piperita* at the H1 temperature without treatment; MpH1M3 = *M.* x *piperita* at the H1 temperature treated with melatonin 3 mM; MpH1SA4 = *M.* x *piperita* at the H1 temperature treated with 4 mM salicylic acid; MpH1M3SA4 = *M.* x *piperita* at the H1 temperature treated with melatonin 3 mM and 4 mM salicylic acid; MpH2C = *M.* x *piperita* at the H2 temperature without treatment; MpH2M3 = *M.* x *piperita* at the H2 temperature treated with melatonin 3 mM; MpH2SA4 = *M.* x *piperita* at the H2 temperature treated with 4 mM salicylic acid; MpH2M3SA4 = *M.* x *piperita* at the H2 temperature treated with melatonin 3 mM and 4 mM salicylic acid; MpH3C = *M.* x *piperita* at the H3 temperature without treatment; MpH3M3 = *M.* x *piperita* at the H3 temperature treated with melatonin 3 mM; MpH3SA4 = *M.* x *piperita* at the H3 temperature treated with 4 mM salicylic acid; MpH3M3SA4 = *M.* x *piperita* at the H3 temperature treated with melatonin 3 mM and 4 mM salicylic acid; MaH1C = *M. arvensis* L. var*. piperascens* at the H1 temperature without treatment; MaH1M3 = *M. arvensis* L. var*. piperascens* at the H1 temperature treated with melatonin 3 mM; MaH1SA4 = *M. arvensis* L. var*. piperascens* at the H1 temperature treated with 4 mM salicylic acid; MaH1M3SA4= *M. arvensis* L. var*. piperascens* at the H1 temperature treated with melatonin 3 mM and 4 mM salicylic acid; MaH2C = *M. arvensis* L. var*. piperascens* at the H2 temperature without treatment; MaH2M3 = *M. arvensis* L. var*. piperascens* at the H2 temperature treated with melatonin 3 mM; MaH2SA4 = *M. arvensis* L. var*. piperascens* at the H2 temperature treated with 4 mM salicylic acid; MaH2M3SA4 = *M. arvensis* L. var*. piperascens* at the H2 temperature treated with melatonin 3 mM and 4 mM salicylic acid; MaH3C = *M. arvensis* L. var*. piperascens* at the H3 temperature without treatment; MaH3M3 = *M. arvensis* L. var*. piperascens* at the H3 temperature treated with melatonin 3 mM; MaH3SA4 = *M. arvensis* L. var*. piperascens* at the H3 temperature treated with 4 mM salicylic acid; MaH3M3SA4 = *M. arvensis* L. var*. piperascens* at the H3 temperature treated with melatonin 3 mM and 4 mM salicylic acid.

### 2.2. Relative Water Content (RWC)

*Relative Water Content* (RWC) was calculated according to Dhopte and Manuel [23]:

RWC = (FW−DW)/(TW−DW) × 100

where FW is leaf fresh weight, DW is dry weight, and TW is leaf turgor mass of leaf samples [24] obtained measuring the leaf weight after 10–12 h in water saturating conditions.

### 2.3. Antioxidant Enzyme Activity

Protein extraction and the activity of antioxidant enzymes(SOD, CAT, GST ans PEROX) was carried out according to Maresca et al. [25].

### 2.4. Isolation of Essential Oils

Samples’ shoots were air-dried in dark conditions at room temperature and were used for essential oils extraction. Each sample (50 g in three replications) was extracted using hydro-distillation for 3 h and Clevenger-type apparatus based on the standard procedure described by Russo et al. [26]. The essential oils were obtained with different yields (0.97 ± 0.02–3.26 ± 0.02%) on dry mass (w/w) and results were yellowish with a pleasant smell. The oils were dried with anhydrous sodium sulfate and stored under N2 at +4 °C in the dark for subsequent tests and analyses.

### 2.5. GC and GC-MS Analysis 

Analytical gas chromatography was carried out on a Perkin-Elmer Sigma 115 gas chromatograph fitted with an Agilent HP-5 MS capillary column (30 m × 0.25 mm), 0.25 μm film thickness. The analysis was also performed by using a fused silica HP Innowax polyethylene glycol capillary column (50 m × 0.20 mm), 0.20 μm film thickness. Gas chromatography analysis was performed as done previously and described in detail by Rigano et al. [27]. Compounds identification and components relative percentages were carried out as described by Rigano et al. [27].

### 2.6. Statistical Analysis 

For each species, the differences between treatments were analyzed by factorial ANOVA (Analysis of Variance) using the hormones and temperature as categorical predictors. The factorial ANOVA was followed by Student-Neuman-Keuls test for post hoc comparisons. Results were reported as mean ± standard deviation.

## 3. Results

### 3.1. Relative Water Content (RWC)

**Figure 1:**
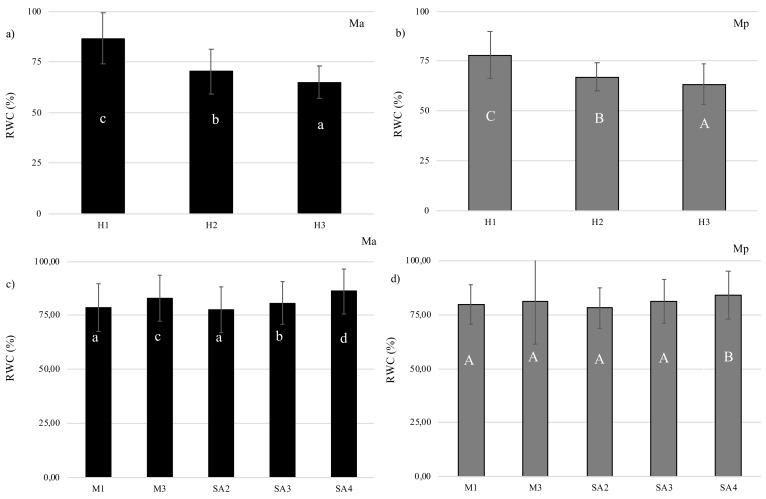
Relative Water Content for (**a**) and (**c**) *M. arvensis* L. var*. piperascens* (Ma) and for (**b**) and (**d**) *M. × piperita* (Mp). Values are presented as means ± standard deviation (n = 15); values not accompanied by the same letter are significantly different at *p* < 0.05, using the post-hoc Student–Newman–Keuls test. Lowercase letters(a–d) indicate significant differences between treatments for Ma; uppercase letters(A–C) indicate significant differences between treatments for Mp.

Significant decrease of Relative Water Contents (RWC%) in H2 and H3 suggested that plants could be under stress. *M. arvensis* L. var*. piperascens* lost more water than *M. × piperita* L. SA and M restored water content in a dose-dependant way (Figure 1). For this reason, for the next experiments, we only used the highest concentration for both treatments: melatonin 30 M (M3) and salicylic acid 4 mM (SA4).

### 3.2. Antioxidant Enzyme Activity

As for the activity of antioxidant enzymes, heating determined a temperature-dependent increase, and treatments with SA4 and M3 determined a further increase, which proved to be extremely significant with the two hormones used simultaneously at their maximum concentrations.

In *M. × piperita*, the activity of all the measured antioxidant enzymes increased with increasing temperature both in the absence and presence of SA and M (M3, SA4). The only exception was the POX activity of samples C, which did not increase with increasing temperature but only under H3 conditions.

Moreover, in most cases the treatment with SA4 had a synergistic effect with the temperature compared to M3 on the activity of all the measured enzymes.

In general, for all the enzymatic activities measured, the samples treated with SA4M3 maintained a significantly higher enzyme activity compared to the C control samples and in the samples treated individually with SA and M3.

In *M. arvensis* L. var*. piperascens* the antioxidant enzymes activity in relation to temperature and treatment with M3, SA4, and M3SA4 followed the same trend shown in *M. × piperita* (Table 1).

### 3.3. Essential Oil Yield

**Figure 2:**
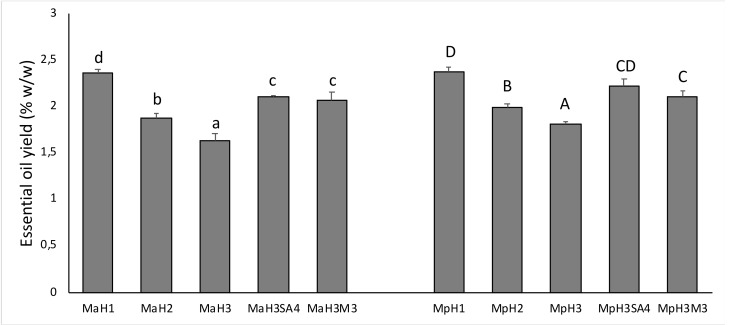
Essential oil yield in *M. arvensis* L. var*. piperascens* (Ma) and *M. × piperita* (Mp) shown in H1, H2, and H3 conditions, and the effect of melatonin (M3) and salicylic acid (SA4) at their highest concentrations on essential oil yield in H3 condition.Values are presented as means ± standard deviation (n = 15); values not accompanied by the same letter are significantly different at *p* < 0.05, using the post-hoc Student–Newman–Keuls test. Lowercase letters(a–d) indicate significant differences between treatments for Ma; uppercase letters(A–D) indicate significant differences between treatments for Mp. For treatments details see Material and Methods Section 2.1).

Essential oil yields in *M. × piperita* and *M. arvensis* L. var*. piperascens* were not statistically different (Figure 2). Heat stress had a similar effect on both species, determining a significant reduction of essential oils as the temperature increased. In addition, both the treatments with SA4 and M3 determined an increase in the yield of essential oils in samples exposed to H3 heat stress conditions, even if there was no statistically significant difference between SA4 and M3.

**Figure 3:**
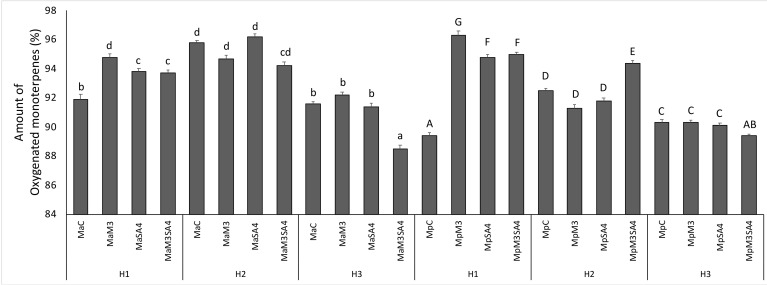
The amount of oxygenated monoterpenes in *M. arvensis* L. var*. piperascens* (Ma) and *M. × piperita* (Mp) under heat stress in H1, H2, and H3 and effects of melatonin (M3) and salicylic acid (SA4) on oxygenated monoterpenes. Values are presented as means ± standard deviation (n = 15); values not accompanied by the same letter are significantly different at *p* < 0.05, using the post-hoc Student-Newman-Keuls test. Lowercase letters(a–d) indicate significant differences between treatments for Ma; uppercase letters(A–G) indicate significant differences between treatments for Mp. For treatment details, see the Material and Methods Section 2.1.

The oxygenated monoterpenes amount in *M. arvensis* L. var*. piperascens* increased by using SA4, M3, and the two of them used simultaneously in normal condition (H1). In H2 conditions, only SA4 increased the oxygenated monoterpenes, while in H3 conditions only M3 increased them. In *M. × piperita* SA, M, and the two hormones used simultaneously increased the oxygenated monoterpenes in H1, and the major effect was observed by using M3. In H2 the oxygenated monoterpenes increased only by using S4 and M3 together. Unfortunately, we could not observe an increase of the oxygenated monoterpenes in H3 (Figure 3). 

A different trend was observed for monoterpene hydrocarbons on respect to oxygenated monoterpenes. In *M. arvensis* L. var*. piperascens* monoterpene hydrocarbons concentration was 4.3% for H1, 3.6% for H2, and 7.4% for H3 in control plants. Their amounts were decreased by using S4 (1.4%), M3 (1.3%), and both simultaneously (S4M3) (1.1%) in H1 conditions. In H2 they decreased by using M3 (0.8%), SA4 (0.8%), and the two of them simultaneously (1.0%), and the same applied for H3 by using SA4 (3.5%), M3 (2.5%) and the two of them simultaneously (4.8%). In *M. × piperita* the monoterpene hydrocarbons concentrations were in H1 4.0%, in H2 3.6% and in H3 5.2% in control plants. In H1 treatment by hormones decreased their amount (about 0.8%), and the same trend of reduction was observed for the other temperature conditions for all the treatments (data not shown).

Oxygenated sesquiterpenes were observed in very low concentrations in both the essential oils. Generally, in *M. arvensis* L. var*. piperascens* and in *M. × piperita* they were reduced or depleted by heat stress. 

**Figure 4:**
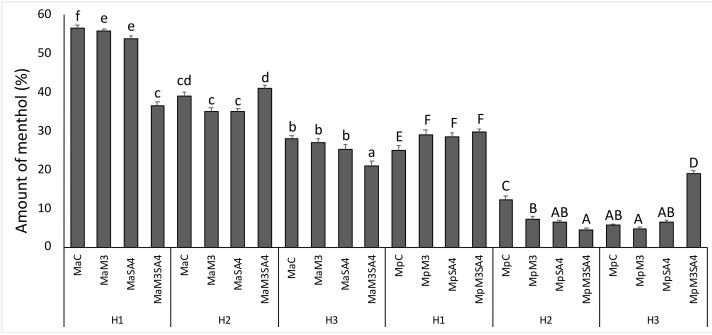
The amount of menthol in *M. arvensis* L. var*. piperascens* (Ma) and *M. × piperita* (Mp) under heat stress in H1, H2, and H3 and effect of melatonin (M3) and salicylic acid (SA4) on menthol. Values are presented as means ± standard deviation (n = 15); values not accompanied by the same letter are significantly different at *p* < 0.05, using the post-hoc Student-Newman-Keuls test. Lowercase letters(a–f) indicate significant differences between treatments for Ma; uppercase letters(A–F) indicate significant differences between treatments for Mp. For treatment details, see the Material and Methods Section 2.1.

The dominant secondary metabolite in mint is menthol. Menthol dramatically decreased by heat stress, more than twice (in H1 56.6%, H2 39.0%, and H3 28.0%) in *M. arvensis* L. var*. piperascens* and about 4.5 fold (in H1 25%, H2 12.2%, and H3 5.6%) in *M. × piperita.* In *M. arvensis* L. var*. piperascens* only using SA4 and M3 simultaneously in H2 conditions the menthol concentration increased. In *M. × piperita* under H1 both SA4 and/or M3 increased the menthol concentration. In H3 by using SA4 and M3 simultaneously, the menthol concentration increased (14.3%) in comparison to control in H3 (Figure 4). 

Menthone is a precursor of menthol. The menthone concentration decreased in *M. arvensis* L. var. *piperascens* in H2 (7.6%) and H3 (12.0%), compared to H1 (14.5%). In *M. × piperita* the menthone concentration increased in H2 (15.9%) and decreased in H3 (9.5%), compared to H1 (13.0%). In normal condition (H1) in *M. arvensis* L. var. *piperascens* M3 decreased and SA4 increased menthone concentration. But in *M. × piperita* all the treatments increased menthone in H1 and H3 conditions. In H2 SA4 and M3 reduced menthone, but using both of them simultaneously increased menthone concentration (data not shown).

The menthofuran concentration in *M. arvensis* L. var*. piperascens* increased (in H1 7.8%, H2 34.0%, H3 13.0%) and in *M. × piperita* decreased (in H1 35.0%, H2 25.4%, H3 34.0%) under heat stress; the highest differences were observed in H2 condition. In *M. arvensis* L. var*. piperascens*, M3 increased, SA4 decreased, and the simultaneous use of both increased the menthofuran concentration, especially in the H1 condition. In H3 all treatments increased the menthofuran concentration. As regards *M. × piperita*, all treatments dramatically decreased the menthofuran concentration in H1. In H2, SA4 and M3 increased the menthofuran concentration. In H3, all the treatments decreased the menthofuran concentration, especially during simultaneous use (data not shown).

**Figure 5:**
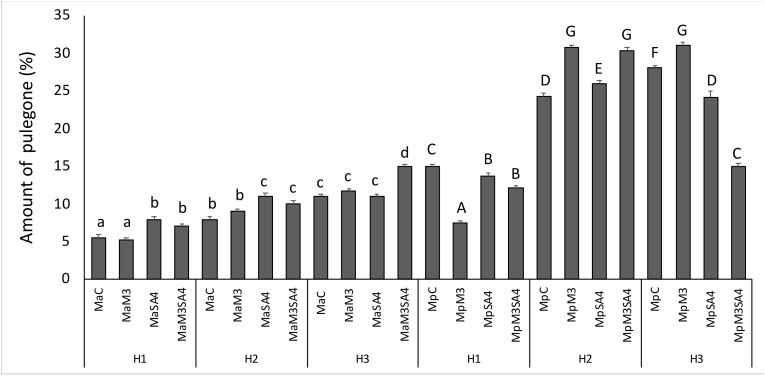
The amount of pulegone in *M. arvensis* L. var*. piperascens* (Ma) and *M. × piperita* (Mp) under heat stress in H1, H2, and H3 and effect of melatonin (M3) and salicylic acid (SA4) on pulegone. Values are presented as means ± standard deviation (n = 15); values not accompanied by the same letter are significantly different at *p* < 0.05, using the post-hoc Student-Newman-Keuls test. Lowercase letters(a–d) indicate significant differences between treatments for Ma; uppercase letters (A–G) indicate significant differences between treatments for Mp. For treatment details, see the Material and Methods Section 2.1.

The pulegone concentrations increased for both the essential oils under heat stress condition. Particularly, the pulegone concentrations in *M. arvensis* L. var*. piperascens* were in H1 5.6%, in H2 8.0%, and in H3 11.0% and for *M. × piperita* in H1 15.0%, in H2 24.3% and in H3 28.1%. In *M. arvensis* L. var*. piperascens* SA4 and the simultaneous use of the two brassinosteroids caused an increase in the pulegone concentration in normal condition (H1). In H2, all treatments increased the pulegone amount. In H3, M3 and the simultaneous use of multiple treatments increased pulegone. In *M. × piperita*, all treatments decreased the pulegone concentration in H1. But as for the heat conditions, in H2 all treatments increased the pulegone amount, while in H3 only M3 increased the pulegone concentration (Figure 5).

In general, menthol has a significant negative correlation with menthofuran (r = −0.459 **) and pulegone (r = –0.912 **), that is to say that menthol is reduced and the pulegone increased under the heat stress. Also, menthofuran had a significant negative correlation with menthone (r = −0.527 **). The pulegone had a significant positive correlation with menthofuran (r = 0.345 *) (Table 2).

## 4. Discussion

In the mid-1980s, the relative water content (RWC) was introduced as the best reference point for the state of water in plants, expressing the balance between water absorption and consumption by transpiration [28]. The heat stress induces an increase of transpiration, with the effect of cooling and adapting the plant to heat. However a further transpiration dries up the tissues and the cells [29]. Generally, osmoregulation is one of the main mechanisms preserving turgor pressure in most plants against water loss; it causes the plant to continue to absorb water and maintain metabolic activity [30]. The RWC leaf may be the best biochemical growth/activity parameter that reveals the severity of stress [31]. Our data show a significant decrease in relative water content (RWC%) in both plants treated with different temperatures, suggesting that both plants were under stress. *M. arvensis* L. var*. piperascens* has lost more water than *M. × piperita* L.

Heat stress disturbs the stable physiological condition in plants and for this reason scientists are trying to find a way to relieve stress. Brassinosteroids such as SA and M have recently been studied in relation to this issue. Coban and Baydar [32] have shown that brassinosteroids reduce salt stress. He et al. [33] inhibited heat stress in bluegrass using SA. In particular, SA stimulates the production and/or an increase of secondary metabolites from polyphenols by acting as an elicitor [34,35]. SA activates phenylalanine ammonia lyase (PAL) [36] and plays a role in the regulation of physiological processes [37]. M also alleviates stress damage, and this has been reported in cucumber in the germination phase [38] and in *Arabidopsis* in which it compensates for heat stress [39]. Our data show that both plants undergo a strong heat stress reducing RWC in a temperature-dependent way. Treatment with SA and M in both plants significantly reduce heat stress effect on RWC, confirming the protective role of these two hormones against heat stress.

In a previous work, we have shown that heat stress determines a change in oxygenated monoterpenes, monoterpene hydrocarbons, oxygenated sesquiterpenes, sesquiterpene hydrocarbons, and other components in *M. × piperita* L. (Mitcham variety) and *M. arvensis* L. (var. *piperascens*) essential oils [22]. In this study, brassinosteroids treatments in both the oils subjected to heat stress determined a variation in the composition of the essential oils and in the antioxidant enzymatic activity. 

Saharkhiz and Goudarzi [40], showed that application of 150 mgL^−1^ SA in *M. × piperita* L. significantly (*p* < 0.05) increased the oil content compared to control plants. In particular the treatment with different SA concentrations mostly increased menthone (15.8–18.1%) and menthol (46.3–47.4%) content. 

**Figure 6:**
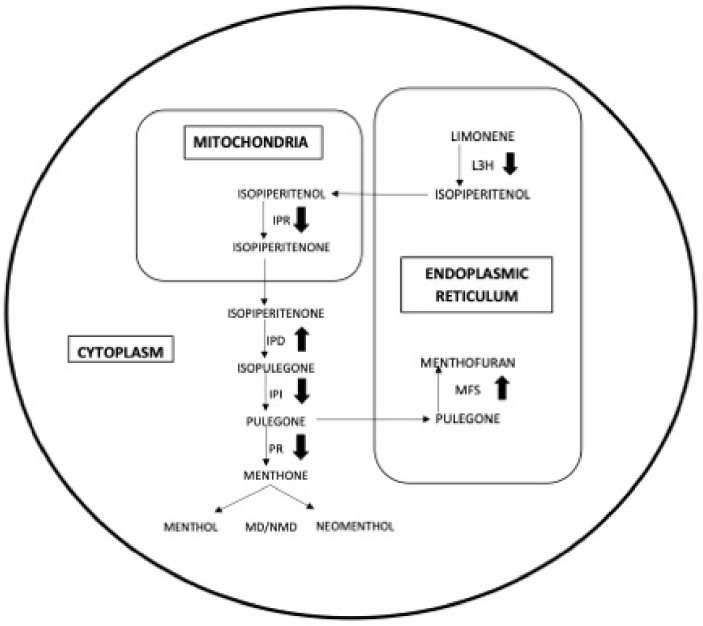
Menthol biosynthesis pathway. IPD: isopiperitenol dehydrogenase, IPR: isopiperitenone reductase, IPI: Isopentenyl diphosphate isomerase, PR: pulegone reductase, LH3:limonene 3-hydroxylase, MFS: menthofuran synthase, NMD: neomenthol reductase, MD: menthol dehydrogenases.

In particular, monoterpenes (synthesized in Methylerythritol phosphate [MEP] pathway) and sesquiterpenes (synthesized in mevalonate [MVA] pathway) were the most important components. In the MEP pathway, menthol is synthesized in the cytoplasm and menthofuran is synthesized in the endoplasmic reticulum [41]. In particular, isopiperitenone from mitochondria is transferred to the cytoplasm and converts to pulegone. Pulegone can continue two branches of the MEP pathway: 1: It remains in the cytoplasm, is converted to menthone and finally to menthol; 2: it is transferred to the endoplasmic reticulum to be converted in menthofuran. So, in the MEP pathway, pulegone, menthone, menthofuran, and menthol have a crucial role and we should consider how they change under heat stress. In general, menthol and menthone have a significant negative correlation with menthofuran and pulegone, (menthol and menthone are reduced and the pulegone and menthofuran increased under the heat stress). Considering the (−)-Menthol biosynthesis pathway (Figure 6), we can hypothesize that under heat stress, pulegone reductase (PR) reduces its activity, leading to a decrease of the conversion of pulegone to menthone (that is the precursor of menthol). The increase of pulegone, due to the reduction of the activity of the enzyme that converts it in menthone, could also explain the increase of the mentofuran, which is synthesized from pulegone in the endoplasmic reticulum. In fact, pulegone had a significant positive correlation with menthofuran (Figure 6). In future studies, it will be necessary to verify the activity of the enzyme PR under heat stress and after treatment with the brassinosteroids. 

As for the antioxidant activity, our studies have shown that SA and M have a positive effect on *M. arvensis* L. var*. piperascens* and *M. × piperita* L., increasing the activity of antioxidant enzymes in both species when used alone, but even more if applied simultaneously, demonstrating a synergistic effect.

On the other hand, an enhanced activity of CAT and SOD was observed in heat stressed plants of *Poa pratensis*, after the treatment with SA [33]. Xu et al. [42] reported that external M applications caused a significant increase in enzymatic antioxidants such as SOD, POX, CAT, and APX peroxidase and non enzymatic antioxidants such as ascorbic acid and vitamin E, resulting in decreased ROS levels and lipid peroxidation in cucumber under high temperature stress. Our data therefore not only confirms the effect of the two hormones on the activity of antioxidant enzymes and therefore the mitigating effect against the heat stress, but also show their ability to act in a synergistic way, which has not been demonstrated so far.

## 5. Conclusions

M and SA alleviate the effects of heat stress in *M. arvensis* L. var*. piperascens* and *M. × piperita* L. by changing the yield of essential oils and the activity of antioxidant enzymes. 

It is possible that the activity of the brassinosteroids highlighted by us occurs through an action by the enzymatic game involved in the metabolism of the studied essential oils. Future studies will aim to highlight a possible modification of enzymatic activity and/or a different expression of the genes involved in their synthesis in response to the presence of M and SA.

Our results can be considered for future applications in the cosmetics, food, and pharmacological fields, given the extreme importance of menthol and menthone in these areas.

## Figures and Tables

**Table 1:** Enzyme activity in *M. × piperita* L. and *M. arvensis* L. var*. piperascens* for each treatment.

	Temperature	Hormons	CAT	GST	POX	SOD
Ma	H1	C	3.188 ± 0.89 ^a^	1.02 ± 0.08 ^a^	19.39 ± 1.25 ^a^	6.88 ± 0.21 ^a^
Ma		M3	34.76 ± 1.94 ^a^	1.78 ± 0.08 ^c^	126.42 ± 1.25 ^c^	12.98 ± 1.03 ^b^
Ma		SA4	42.56 ± 2.05 ^a^	1.49 ± 0.06 ^b^	98.12 ± 3.69 ^c^	10.33 ± 1.05 ^bc^
Ma		M3SA4	66.42 ± 2.45 ^d^	2.54 ± 0.06 ^d^	226.77 ± 5.11 ^d^	18.45 ± 1.13 ^bc^
Ma	H2	C	9.46 ± 1.83 ^b^	1.52 ± 0.03 ^d^	22.73± 1.04 ^a^	9.98 ± 1.01 ^c^
Ma		M3	63.86 ± 2.95 ^c^	2.01 ± 0.08 ^e^	245.13 ± 9.46 ^d^	43.79 ± 1.13 ^d^
Ma		SA4	98.73 ± 2.83 ^b^	2.67 ± 0.08 ^h^	108.42 ± 3.14 ^f^	19.07 ± 1.16 ^e^
Ma		M3SA4	134.52 ± 2.54 ^e^	4.29 ± 0.08 ^i^	269.67 ± 1.22 ^g^	54.53 ± 1.51 ^f^
Ma	H3	C	19.61 ± 1.67 ^d^	2.28 ± 0.02 ^f^	4.96 ±1.45 ^b^	16.88 ± 0.71 ^d^
Ma		M3	92.37± 2.04 ^e^	2.67 ± 0.04 ^g^	316.91± 7.70 ^e^	49.07 ± 0.73 ^g^
Ma		SA4	129.04 ± 3.75 ^e^	3.74 ± 0.06 ^l^	146.95 ± 4.22 ^h^	49.12 ± 0.87 ^g^
Ma		M3SA4	130.33 ± 2.16 ^f^	4.61 ± 0.09 ^m^	491.12 ± 11.77 ^i^	58.77 ± 0.89 ^h^
Mp	H1	C	9.53 ± 1.33 ^a^	0.92 ±0.04 ^a^	11.49 ± 1.25 ^a^	5.64 ± 0.55 ^a^
Mp		M3	10.51 ± 1.07 ^a^	1.70 ± 0.03 ^c^	61.50 ± 1.42 ^c^	18.61 ± 1.62 ^b^
Mp		SA4	12.54 ± 1.46 ^a^	1.57 ± 0.07 ^b^	66.26 ± 2.38 ^c^	20.61 ± 0.83 ^bc^
Mp		M3SA4	41.15 ± 2.03 ^d^	1.93 ± 0.1 ^d^	103.57 ±5.45 ^d^	20.54 ± 0.53 ^bc^
Mp	H2	C	19.42 ± 1.47 ^b^	1.93 ± 0.03 ^d^	15.19 ± 1.52 ^a^	22.06 ± 0.90 ^c^
Mp		M3	29.54 ± 1.19 ^c^	2.28 ± 0.07 ^e^	95.12 ± 1.20 ^d^	28.75 ± 2.23 ^d^
Mp		SA4	22.39 ± 1.54 ^b^	3.12 ± 0.08 ^h^	154.46 ± 4.56 ^f^	33.48 ± 0.82 ^e^
Mp		M3SA4	54.49 ± 2.50 ^e^	3.74 ± 0.07 ^i^	343.70 ± 6.38 ^g^	51.21 ± 1.31 ^f^
Mp	H3	C	42.75 ± 1.6 ^d^	2.63 ± 0.04 ^f^	36.79 ± 0.82 ^b^	29.81 ± 1.19 ^d^
Mp		M3	53.12 ± 2.04 ^e^	2.99 ± 0.08 ^g^	138.90 ± 7.00 ^e^	56.40 ± 1.47 ^g^
Mp		SA4	51.76 ± 2.26 ^e^	6.13 ± 0.08 ^l^	417.25 ± 13.36 ^h^	54.50 ± 1.52 ^g^
Mp		M3SA4	130.33 ± 2.16 ^f^	6.52 ± 0.08 ^m^	466.18 ± 16.72 ^i^	66.61 ± 1.27 ^h^

Values are presented as means ± standard deviation (n = 15); values not accompanied by the same letter(^a–i,l,m^), are significantly different at *p* < 0.05 using the post-hoc Student–Newman–Keuls test. For treatment details, see the Material and Methods Section 2.1.

**Table 2:** Pearson correlation coefficients found among four important secondary metabolites (menthofuran, menthol, pulegone, and menthone) in the menthol pathway in *M. arvensis* L. var*. piperascens* and *M. × piperita* under the long-term extreme heat stress by using SA and M as a compensator of stress.

	Menthofuran	Menthol	Pulegone	Menthone
Menthofuran	1	−0.459 ^**^	0.345 ^*^	−0.527 ^**^
Menthol		1	−0.912 ^**^	−0.054
Pulegone			1	0.009
Menthone				1

** Correlation is significant at the 0.01 level (2-tailed).* Correlation is significant at the 0.05 level (2-tailed).
